# A Commentary on Physico-chemical and Biological Activity of Biogenic Amines and Their Relationship with Safety of Cheese

**DOI:** 10.3389/fmicb.2012.00228

**Published:** 2012-07-05

**Authors:** Antonio Valero

**Affiliations:** ^1^Department of Food Science and Technology, University of CórdobaCórdoba, Spain

This manuscript offers a comprehensive review about the bacterial amino acid decarboxylase activity and biogenic amines content of an artisanal cheese produced in Italy, named as Pecorino cheese. It is highlighted the importance of controlling primary production of raw milk, as well as cheese-making process to avoid the release of free amino-acids and viability of microorganisms possessing amino acid decarboxylating activity. Variation in other physico-chemical parameters, such as water activity or protein content, or during ripening may produce the selection of different microbial populations which could increase the formation of biogenic amines in the final product. Among the biogenic amines described in the manuscript, the formation of diamines (putrescine and cadaverine) and specially, tyramine content resulted to be present in high proportion in most of Pecorino cheeses. In some cases, mild thermal treatments applied in milk appear not to be sufficient to eliminate decarboxylase-positive bacteria in cheeses. Additionally, proteinases and peptidases released from rennet could be responsible of high content of biogenic amines. The authors concluded that the formation of biogenic amines depends on several factors and it would be necessary to study case-by-case each cheese type given the high variability in physico-chemical composition. The quality of raw milk, processing conditions, ripening, and subsequent storage before consumption may influence the biogenic amines formation. There are not so many studies made in this area and this could be particularly interesting in other cheese types, where artisanal production is commonly followed.

